# Meiotic prophase I defects in an oligospermic man with Wolf-Hirschhorn syndrome with ring chromosome 4

**DOI:** 10.1186/1755-8166-7-45

**Published:** 2014-07-01

**Authors:** Qi Yao, Liu Wang, Bing Yao, Hongliu Gao, Weiwei Li, Xinyi Xia, Qinghua Shi, Yingxia Cui

**Affiliations:** 1 Institute of Laboratory Medicine, Jinling Hospital, Nanjing University School of Medicine, 305 East Zhongshan Road, Nanjing 210002, PR China; 2 Institute of Reproductive Medicine, Jinling Hospital, Nanjing University School of Medicine, 305 East Zhongshan Road, Nanjing 210002, PR China; 3 Laboratory of Molecular and Cell Genetics, Hefei National Laboratory for Physical Sciences at Microscale and School of Life Sciences, University of Science and Technology of China, Hefei, China

**Keywords:** Ring chromosome 4, Oligospermia, Synapse complex, Recombination, Transcriptional inactivation

## Abstract

**Background:**

Ring chromosomes are often associated with spermatogenetic failure. However, the mechanism is poorly understood. We here reported a single man with severe oligospermia and a ring chromosome 4 with a microdeletion at 4p16.3.

**Results:**

Synapsis (as SCP3), recombination (as MLH1) and transcriptional inactivation (as BRCA1) in a testicular biopsy were examined by fluorescence immunostaining. In the oligospermia patient, 35.4% of spermatocytes were in zygotene phase compared with 5.2% in controls. The patient had a significantly reduced recombination frequency with mean of 45.9 MLH1 foci/cell compared with 47.8 in controls. In the patient, chromosome 4 in all pachytene cells displayed loop formation with varying degrees of unpaired regions. BRCA1 localized along asynapsed regions regardless of XY body association.

**Conclusions:**

Ring chromosome 4 might affect the progression of meiosis I prophase, synapse formation, and transcriptional activation of asynapsed areas, and impair male fertility.

## Background

Chromosomal structural abnormalities such as inversion, translocation and complex chromosome rearrangements can disturb the first meiotic division and result in sterility [1-4]. Ring chromosomes are rare chromosomal structural abnormalities. The phenotypes of patients with ring chromosome include physical and mental defects due to loss of genomic material and ring formation [5], as well as spermatogenic arrest [6-16]. However, the underlying mechanisms of spermatogenetic failure in patients with ring chromosomes are not fully understood. Here, we report a patient with ring chromosome 4 and microdeletion of chromosome region 4p16.3 presenting the core features of Wolf-Hirschhorn syndrome and spermatogenic arrest. To understand the mechanisms of spermatogenic arrest in this patient, this study used fluorescence immunocytogenetic methods to investigate the progression of meiosis I prophase, chromosome pairing and recombination, transcriptional inactivation.

## Case presentation

### Patient

**Figure 1:**
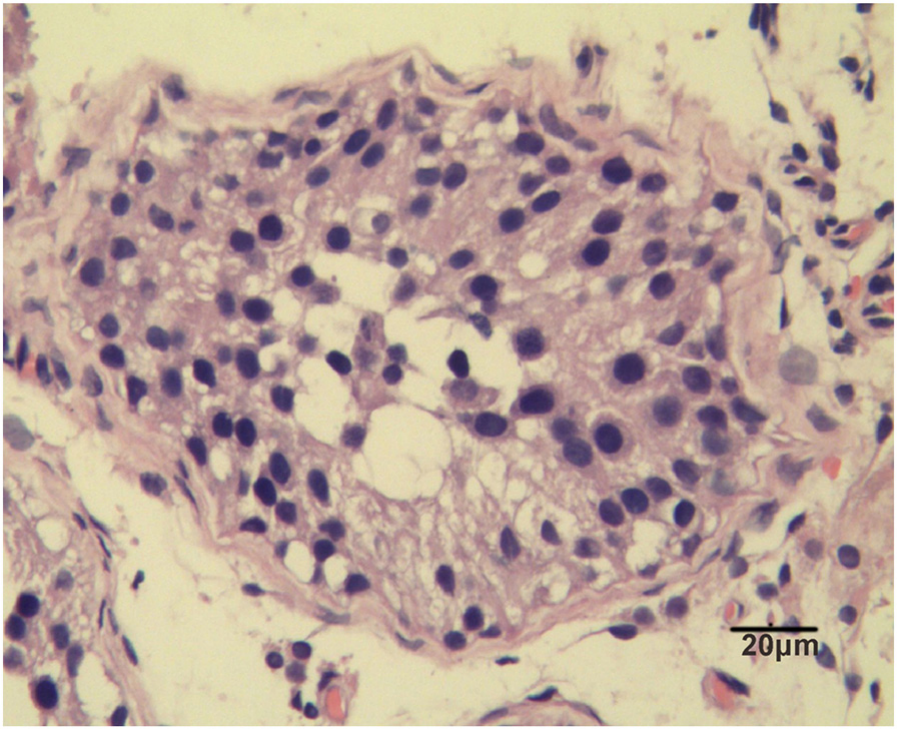
**Histological analysis of testicular tissue from the patient with a ring chromosome 4 and microdeletion of 4p16.3.** Testicular tissue demonstrates reduced number of primary spermatocytes and spermatogenic arrest at the primary spermatocyte stage.

The patient was a 23-year-old man who presented at our center for oligospermia. The patient showed signs of Wolf-Hirschhorn syndrome. He had left-side cryptorchidism. His right scrotal testis was slightly soft and small (12 mL; normal testes size for adult Chinese 19.8 ± 7.1 mL). His penis was appropriate in size. Semen analysis revealed a semen volume of 1 ml, sperm concentration of 2.81 million per ml, and motility of 14.63%, with a total motile sperm count of 0.41 million. A repeat sample 2 weeks later showed a semen volume of 1 ml, sperm concentration of 0.73 million per ml, and motility of 12%, with a total motile sperm count of 0.09 million. A CT brain scan found no abnormality. Abdominal ultrasound revealed no visceral anomalies. Intelligence was in the lower limit of the normal range. After obtaining informed content from the patient and his parents, biopsy was performed on the right testis. Histology of the testicular biopsy sample showed the number of primary spermatocytes was reduced, and spermatogenic arrest at the primary spermatocyte stage (Figure 1).

Hormonal profile was normal for serum concentrations of PRL, LH, and T. FSH concentration was higher than normal (18.9 IU/L, normal value 1.0-5.5 IU/L). Y-chromosome screening for AZF microdeletions was normal.

**Figure 2:**
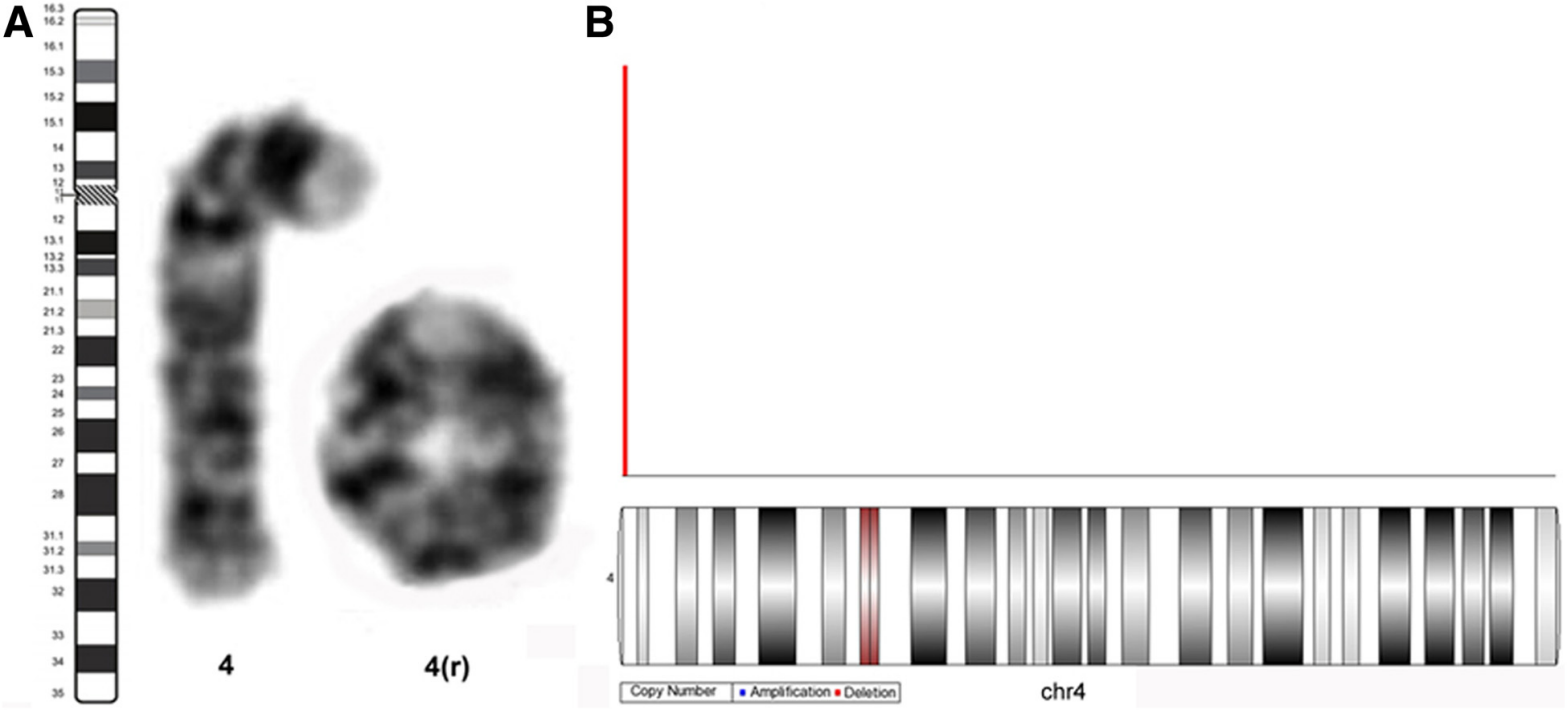
**Karyotype analysis. (A)**: Partial G-banded karyotype of peripheral blood lymphocytes showing a normal chromosome 4 and ring chromosome 4. **(B)**: Array CGH analysis of chromosome 4 in the patient with microdeletion at 4p16.3.

Karyotype analysis of peripheral blood lymphocytes by classic G-binding revealed 46,XY,r(4)(::p16.3 → q35.2::)[31]/45,XY,-4[3] (Figure 2A). To characterize the ring chromosome 4, molecular cytogenetic analysis was performed using the Affymetrix Genome-wide Human SNP array 6.0 platform (Affymetrix Inc., Santa Clara, CA). Array data were analyzed using Genotyping Console software. The results indicated a terminal 852 kb deletion at 4p16.3 that included 17 annotated genes (MIRN571, LOC654254, ABCA11, LOC642707, ZNF141, ATP51, GAK, ZNF595, MGC26356, ZNF718, MFSD7, PDE6B, PCGF3, MYL5, PIGG, ZNF721, and CPLX1) (Figure 2B). Thus, the final karyotype was 46,XY,r(4)(::p16.3 → q35.2::)dn.arr[hg18]4pterp16.3(57,770-910,354) × 1[31]/45,XY,-4[3]. Parental karyotypes were normal, indicating *de novo* origin of the ring chromosome 4.

## Materials and methods

### Meiotic analyses

Testicular tissue was processed as described previously [17]. Control samples C1-C5 were obtained from five prostate cancer patients with proven fertility who had undergone orchiectomy (age range: 64–69 years). Informed consent was obtained from the patient, his parents and the five control patients, and research was approved by the Ethics Committee of the Nanjing Jinglin Hospital.

Primary antibodies were rabbit anti-SCP3 (donated by Dr Christa Heyting, University of Wageningen, The Netherlands), human anti-CREST (Immunovision, Springdale, AR), mouse anti-MLH1 (BD Pharmigen Bioscience, San Diego, CA), and mouse anti-BRCA1 (Santa Cruz Biotechnology, Santa Cruz, CA). Primary antibodies were detected using secondary antibodies Alexa 555 donkey anti-rabbit (Molecular Probes, Carlsbad, CA), Alexa 488 goat anti-mouse (Molecular Probes), and 1-amino-4-methylcoumarin-3-acetic acid (AMCA) donkey anti-human (Jackson Immunoresearch, West Grove, PA).

Cell evaluation and image capturing were by epifluorescence microscopy (Olympus BX61, Olympus Inc., Tokyo, Japan) and Image Pro-Plus version 5.1 software (Media Cybernetics Inc., Bethesda, MD). The progression of meiotic prophase I were distinguished by the appearance and chronology of synaptonemal complex (SC) protein SCP3. Multiple short SCP3-positive segments were revealed at leptotene; 46 complete but unpaired SCP3-positive elements with 46 CREST signals (a mark of centromere) were observed at zygotene; synapsis of homologues was completed with 23 CREST, 23 SCs signals and the appearance of MLH1 foci (a mark of meiotic recombination sites) at pachytene.

Statistical analysis used SPSS 16.0 software (SPSS Inc., Chicago, IL). The Mann–Whitney test was used to compare MLH1 foci per cell between the patient and controls. A chi-square test was applied to compare recombination rates of XY pairs in the patient and controls.

## Results

Meiotic prophase I substages were identified in the patient's biopsy sample using immunofluorescence to determine the appearance and chronology of SCP3. 164 primary spermatocytes from the patient and 1828 from five control men were analyzed. For the five controls, mean frequencies of cells by stage were 13.8% for leptotene, 5.2% for zygotene, and 81% for pachytene. In the patient, 35.4% of cells were in zygotene stage, indicating blockage at this stage; with 12.2% of cells in leptotene stage, and 52.4% in pachytene stage.

**Figure 3:**
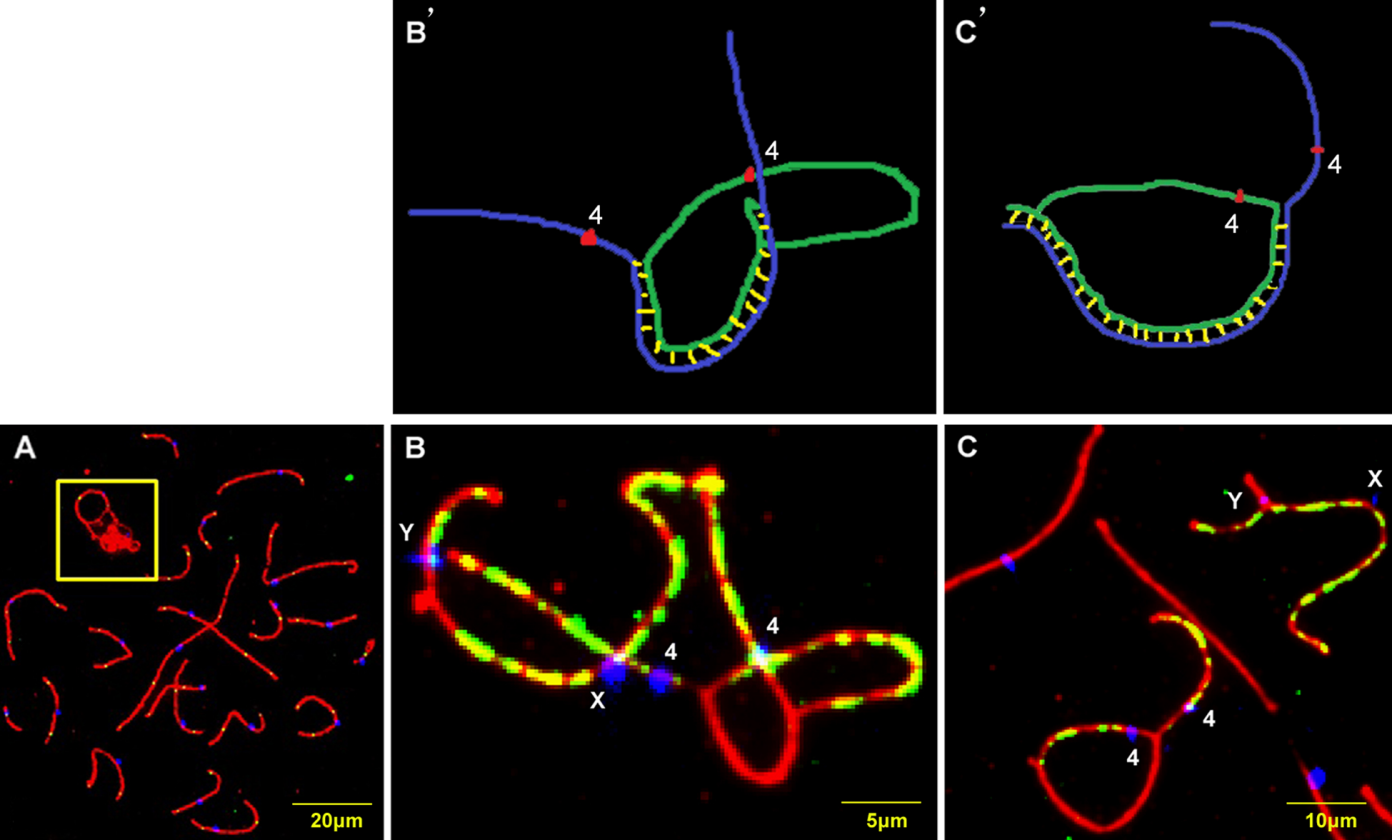
**Fidelity of synapsis and transcriptional inactivation in a ring chromosome 4. (A)**: Immunostaining of synaptonemal complexes (SCP3, red), centromeres (blue), and recombination sites (MLH1, green) in spermatocyte from the patient with a ring chromosome 4 with microdeletion at 4p16.3. Spermatocyte shows asynapsed ring with two free ends (box). **(B’-C’)**: Schematic representation of chromosome 4 indicating the asynapsed and synapsed parts of it. Linear chromosome 4 is shown in blue, ring chromosome 4 in green, synapsed area in yellow, and centromeres in red. **(B-C)**: Immunolocalization of BRCA1 (green), centromeres (blue), and SCP3 (red) in spermatocytes of the patient. Sex chromosomes (excluding psedoautosomal region) and asynapsed area of chromosome 4 display BRCA1 labeling. **(B and B’)**: Part of a spermatocyte with an incompletely synapsed ring associated with sex chromosomes. **(C and C’)**: Part of a spermatocyte with an incompletely synapsed ring not associated with sex chromosomes.

For the patient and for each of the five controls, recombination frequencies were determined for 73 pachytene nuclei. In the patient, the mean number of MLH1 foci per cell was significantly lower than the number in controls (45.9 ± 5.9 vs. 47.8 ± 4.5 *P* = 0.004, Mann–Whitney test). However, the recombination frequency in the patient, as determined by XY bivalents, was not significantly different from the controls (76% vs. 78%, *P* = 0.731, chi-square test).In all pachytene nuclei analyzed from the patient, the chromosome 4 homologs paired and formed loops with two free ends. The loops showed different degrees of asynapsis between the homologs (Figure 3).In pachytene nuclei of the patient carrying ring chromosome 4, BRCA1 staining was seen in the X and Y axis (excluding the pseudoautosomal region) and in the asynaptic segments of the axes of chromosome 4 (Figure 3B-C).

## Discussion

Wolf-Hirschhorn syndrome is a congenial anomaly due to a deletion in the distal short arm of chromosome 4. The main clinical picture is characterized by dysmorphic facial features, delayed growth, delayed psychomotor and multiple malformations [18-20]. The syndrome was often diagnosed at early life. However, in this case, the diagnosis has not been made until adulthood, which let us find a novel phenotype of spermatogenic arrest.

A series of complex processes involving pairing, synapsis, recombination, and segregation of homologous chromosomes take place during the first meiotic division. When any of these processes is altered, cellular checkpoints will arrest the progression of meiosis and induce cell loss, which causes a severe reduction in fertility, or even sterility. The main characters of spermatogenesis in the present patient were synapse defect between the homologs of chromosome 4 and pachytene arrest. The conditions confirmed synapse defect might trigger meiosis checkpoints and lead to spermatogenic arrest at the pachytene stage and finally, resulting in spermatogenesis failure. Three checkpoints are reported to be responsible for meiotic arrest at the zygotene-pachytene transition, including DNA double-strand breaks (DSBs), SC and meiotic inactivation of sex chromosome (MSCI) [21-24]. The operation of all these three checkpoints can be triggered by errors in chromosomes synapsis (also called asynapsis) when asynapsed chromosome segments reach a threshold. As the configuration of loops varied from cell to cell, different checkpoints might be triggered in different spermatocytes even in the same patient. Therefore, for the patient, unpaired fragments in most primary spermatocytes reached the threshold, displaying spermatogenic arrest at the pachytene stage, while unsynapsis segments in a few cells may be too small to be detected by the pachytene checkpoints, revealing a phenotype of oligozoospermia.

In mammals, asynapsis in males is almost always associated with spermatocyte losses during the pachytene stage of meiotic prophase and/or at the metaphase stage of the first meiotic division, with consequent subfertility or sterility [22]. It has been proposed that unsynapsed chromosomal regions could drive a process called meiotic silencing of unsynapsed chromatin (MSUC), which may disrupt the normal loading of MSUC proteins, interfere with autosome and sex chromosome gene expression [21,22] and trigger a massive pachytene cell death. In our case, synapse defect of the homologs of chromosome 4 was linked to reduction of primary spermatocytes in seminiferous tubular. The mechanism of reproductive impairment might be ascribed to MSUC and further lead to pachytene apoptosis.

Compared with control patients, a significant reduction in the mean number of MLH1 foci per cell was found in our patient, however, the mean MLH1 foci per cell number of our patient was still within the range of normal men [25,26] shown in the previous literature. Thus, the decrease of MLH1 foci per cell in our patient might be due to interindividual variation, but not donor age and status [27]. Indeed, several reports described that differences in recombination could be explained by the presence of SNPs in genes involved in meiotic recombination. [28,29]. Therefore, it was difficult to assess the role of less MLH1 foci during the process of spermatogenesis in our patients.

It has been proposed that a low frequency of recombination in the psedoautosomal region may associate to infertility in azoospermic and severe oligospermic patients who did not carry a reorganization [26]. While there is no significant difference between our patient and the controls in terms of XY recombination frequency, which may support our hypothesis that the presence of the ring chromosome influences the meiosis of the patient.

The ring chromosome 4 had a 852 kb deletion at 4p16.3. No direct evidence from the literature indicates that the 17 deleted genes at 4p16.3 are associated with spermatogenesis. The functions of some the genes are still unknown. Therefore, the possibility that the 17 genes on 4p16.3 resulted in spermatogenic arrest could not be excluded.

In addition, some other potential factors may interfere with spermatogenesis for the patient, such as deleterious effect of left cryptorchidism [30], or presence of interchromosomal effect (ICE) [22] due to carrying a ring chromosome 4. However, no direct evidences in our experiments were found from the patient. The molecular mechanism should be further investigated.

## Conclusion

In summary, we report a ring chromosome 4 with a 852 kb deletion at 4p16.3 that might affect the progression of meiosis I prophase, synapsis, and transcriptional activation of asynapsed areas, leading to spermatogenic arrest. Additional cases and future research will determine if a single deleted gene, a cluster of genes deleted at 4p16.3, or the chromosome structural abnormality contribute to impaired spermatogenesis.

## Competing interest

The authors declare that they have no competing interests.

## Authors’ contributions

QY, LW and HLG played a role in biological experiments and molecular diagnosis. BY and YXC had major roles in patient and control recruitment, collection and analysis of clinical data. All authors contributed to the writing and revision of the manuscript.
